# Transitioning from PET/MR to trimodal neuroimaging: why not cover the temporal dimension with EEG?

**DOI:** 10.3934/Neuroscience.2023001

**Published:** 2023-02-20

**Authors:** Arosh S. Perera Molligoda Arachchige

**Affiliations:** Department of Biomedical Sciences, Humanitas University, Milan, Italy

**Keywords:** PET, MR, EEG, multimodality, neuroimaging

## Abstract

The possibility of multimodality imaging with PET/MR and the availability of ultra-high field MRI has allowed to investigate novel aspects of neuropsychiatric conditions. One of the major barriers in current studies is the lack of an instrument that allows to accurately cover the temporal aspect under the same physiological conditions. The aim of this commentary is to provide our perspective on how the integration of EEG-PET-MR could be a solution to the current challenge in molecular imaging and seems to hold great promise in future pharmacological challenging-based studies, understanding different functional states of the brain, and could furthermore aid in the diagnostic and prognostic evaluations of neurocognitive disorders.

With the advent of ultra-high field imaging and integrated PET/MRI systems within the last decade, it seems that the field of neuropsychiatric research has unlocked the potential to reach new heights. This is because these ultra-high field MR-PET systems are known to yield superb spatial resolution along with highly specific molecular data [1]. However, in the study of neurocognitive disorders with fMRI, the lack of an accurate temporal aspect revealing instrument in the sub-second range had since recently been a barrier. This is where the possibility of a triple modality system comes into play.

Indeed, a study by Del Guerra et al. describe this type of EEG system appended to a 1.5T PET/MRI implemented with success within the framework of the EU project TRIMAGE and has been shown to allow comprehensive understanding of brain function (See Fig.1) [2]. This is because EEG electrode-mediated PET signal attenuation is negligible and PET electronics do not interfere with electrophysiological signals. Moreover, the EEG signal can reflect functional changes allowing investigation of brain dynamics since it can be modulated by pharmacological manipulations [3]. Furthermore, the integration of EEG makes it possible to overcome one of the major drawbacks of fMRI which uses the BOLD effect to indirectly measure brain function. This is because the delayed hemodynamic response decreases the temporal resolution of the BOLD effect [4]. This triple modality combination holds great promise in the investigation of pharmacological challenging-based emotional, social, and cognitive paradigms owing to the elimination of learning and order effects [3]. In addition, it also allows to investigate and define different functional states of the brain. Multimodality systems maximise patient comfort and are timesaving rendering them more compliant and reduce dropout during follow-up in clinical research [1]. For example, the device developed by the TRIMAGE project is cheaper and is not complex to maintain due to the absence of either liquid nitrogen or liquid helium, and additionally it is equipped by an MR magnet which is axially shorter leaving the patients shoulders outside ultimately minimizing claustrophobic effects [2]. Above all, it makes possible to maintain same physiological conditions for both datasets.

What could be the possible clinical applications of such a trimodal system? For example, in a study by Golkowski et al. EEG/fMRI/PET was used to investigate brain activity in a small sample of patients with disorders of consciousness which they refer to as an approach that provides multiple markers of consciousness under the same physiological conditions, enabling a more accurate diagnosis and better estimation of prognosis which would be otherwise difficult to categorize [9]. The triple modality approach might also be valuable for investigating the fundamentals of brain function, for example, resting state networks that underpin many processes can be altered in disease states. A recent work by Régio Brambilla et al. showed that event related potential components that are affected by disease states and disorders can also be useful in this trimodal approach. In their study, task-induced changes in glutamatergic neurotransmission were found using the mismatched negativity paradigm which provides further evidence that EEG-fMRI-PET is a useful approach for investigating the role of glutamatergic neurotransmission in both the healthy and subjects suffering from disorders such as dementia and schizophrenia [10].

Nonetheless, implementation of such a system does not come without constraints. Great care is needed when recording data to maintain an acceptable level of data quality and to ensure patient safety during the procedure. There are also technical challenges due to artefacts, one of which is the “cardioballistic artefact” occurring due to cardiac pulsatile activity causing pulsation of scalp vessels and head movements [5]. The other, caused by the switching of gradients during the preferred sequence for fMRI, the echo planar imaging sequence giving rise to the so-called “gradient artefact” [6]. Moreover, there are artefacts related to the currents induced due to the movement of conductive materials including the EEG electrodes and cables within the magnetic field also given that the scanner cryogen pump as well as gradient switching produces vibrations, and furthermore due to the inevitable motion of the study subject [7]. Even though there exist studies claiming that these artefacts may be removed with success through techniques such as Independent Component Analysis, Artefact Average Template Subtraction, etc. the real problem is that some of these artefacts have been shown to linearly increase with the increases in field strength and thus becoming more challenging to remove with their integration into ultra-high field scanners [8]. Lastly, there is the issue of integrating the different acquisition timeframes.

Despite all the aforementioned technical and practical challenges in simultaneous PET/MR/EEG, richness of the complementary data obtained through numerous studies have elucidated that it is of potential added value in the diagnosis, treatment, and monitoring of neuropsychiatric disorders. Thus, we believe that with multimodal data integration through the use of machine learning methods and appropriate techniques to overcome these challenges encountered could in future reveal novel aspects of neuropsychiatric disorders [11].

**Figure 1.**
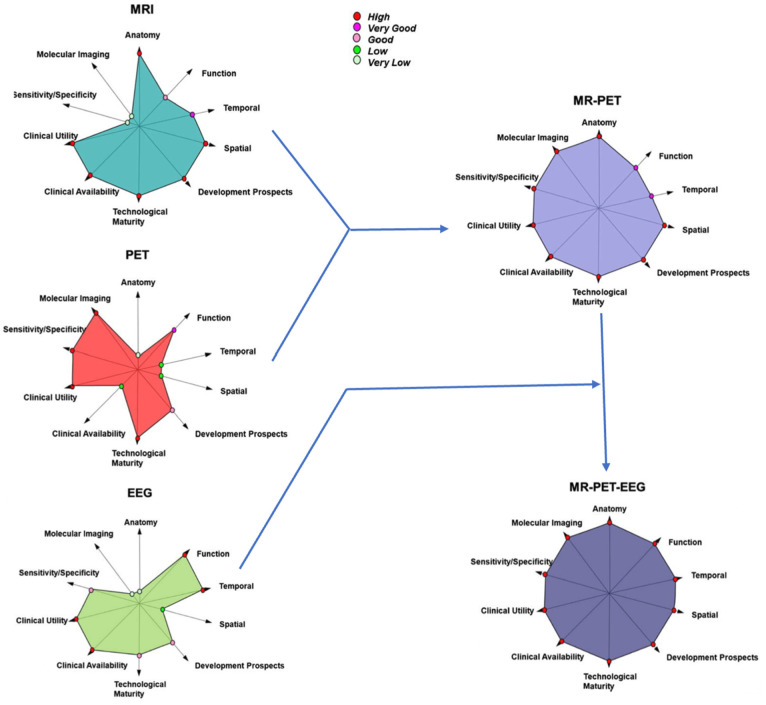
Diagrams pertaining to each of MRI, PET, PET/MRI, PET/MRI/EEG, showing the strengths of the relevant attributes presented along each axis. The further the point is from the centre, the higher is the strength of the relevant attribute. Notice how the integration of EEG to the PET/MRI markedly strengthens temporal mapping of brain function.
